# Significance of circadian rhythms in severely brain-injured patients: A clue to consciousness?

**DOI:** 10.1212/WNL.0000000000003942

**Published:** 2017-05-16

**Authors:** Christine Blume, Julia Lechinger, Nayantara Santhi, Renata del Giudice, Maria-Teresa Gnjezda, Gerald Pichler, Monika Scarpatetti, Johann Donis, Gabriele Michitsch, Manuel Schabus

**Affiliations:** From the Laboratory for Sleep, Cognition and Consciousness Research, Department of Psychology (C.B., J.L., R.d.G., M.-T.G., M. Schabus), and Centre for Cognitive Neuroscience Salzburg (CCNS) (C.B., J.L., R.d.G., M. Schabus), University of Salzburg, Austria; Surrey Sleep Research Centre (N.S.), Faculty of Health and Medical Sciences, University of Surrey, UK; Albert Schweitzer Clinic (G.P., M. Scarpatetti), Apallic Care Unit, Geriatric Health Centres of the City of Graz; and Neurologische Abteilung mit Wachkomabetreuung (J.D., G.M.), Pflegewohnhaus Donaustadt Wien, Vienna, Austria.

## Abstract

**Objective:**

To investigate the relationship between the presence of a circadian body temperature rhythm and behaviorally assessed consciousness levels in patients with disorders of consciousness (DOC; i.e., vegetative state/unresponsive wakefulness syndrome or minimally conscious state).

**Methods:**

In a cross-sectional study, we investigated the presence of circadian temperature rhythms across 6 to 7 days using external skin temperature sensors in 18 patients with DOC. Beyond this, we examined the relationship between behaviorally assessed consciousness levels and circadian rhythmicity.

**Results:**

Analyses with Lomb-Scargle periodograms revealed significant circadian rhythmicity in all patients (range 23.5–26.3 hours). We found that especially scores on the arousal subscale of the Coma Recovery Scale–Revised were closely linked to the integrity of circadian variations in body temperature. Finally, we piloted whether bright light stimulation could boost circadian rhythmicity and found positive evidence in 2 out of 8 patients.

**Conclusion:**

The study provides evidence for an association between circadian body temperature rhythms and arousal as a necessary precondition for consciousness. Our findings also make a case for circadian rhythms as a target for treatment as well as the application of diagnostic and therapeutic means at times when cognitive performance is expected to peak.

We are governed by manifold rhythmic processes that affect our body at all levels from gene expression to higher cognitive functions.^1–3^ Many of these processes follow a circadian pattern; that is, they have a period length of approximately 24 hours and are under tight control of a biological master clock located in the suprachiasmatic nuclei of the hypothalamus.^4,5^ Moreover, rhythms spanning all levels of physiology and behavior are well-orchestrated and, thus, often strongly coupled. For example, Wyatt et al.^6^ found variations in cognition to parallel the circadian temperature rhythm such that alertness and performance peaked around the core body temperature maximum (i.e., at about 4 pm in the average healthy day-active person). Subsequent studies have confirmed this relationship, although its magnitude seems to be task-dependent (see reference 7 for a review).

Given the circadian variations in global states like alertness, it is not surprising that consciousness also varies rhythmically in healthy individuals. One very prominent example of this rhythm is the sleep-wake cycle, during which consciousness fades and recovers on a diurnal basis. With regard to prevailing theories of consciousness, it can be speculated that circadian variations also directly affect the brain's ability to (1) integrate information (information integration theory)^8,9^ or (2) modify the likelihood that neuronal activation results in ignition and broadcasting of information to the whole brain (global neuronal workspace model).^10^ In fact, this notion is supported in a recent study by Ly et al.,^11^ who report circadian modulation of cortical excitability, a background condition for consciousness.

From a clinical perspective, misalignment of circadian rhythms, which occurs when the sleep–wake schedule is at odds with the light–dark cycle as in the case of night shifts, can cause considerable stress, have detrimental effects on the immune system, and impair cognitive abilities such as attention and learning.^12,13^ Moreover, temporal disorganization of circadian rhythms, i.e., the uncoupling of different rhythms, has been shown to have pathologic significance in critically ill patients and it has been suggested that this may hinder recovery.^14,15^ Despite the knowledge that entrained circadian rhythms are important for healthy body and brain functioning, very little is known about circadian rhythms in patients diagnosed with a disorder of consciousness (DOC) following a severe brain injury. DOC states comprise the vegetative state (VS, also referred to as unresponsive wakefulness syndrome [UWS]) and the minimally conscious state (MCS). While patients in VS/UWS present periods of wakefulness with eye opening and sleep, they are presumably unconscious. Patients in MCS, in contrast, present inconsistent but identifiable signs of conscious awareness.^16^ When patients in MCS recover the ability to functionally interact with their environment, they are classified as exit MCS.^17^

Studying circadian rhythms in DOC patients may be especially interesting and important for several reasons. First, the presence or absence of circadian rhythms as well as anomalies in them could be informative about the state of the patient as well as the potential for recovery. Second, this could provide information about time points that best capture remaining cognitive functions, e.g., with behavioral scales such as the Coma Recovery Scale–Revised (CRS-R),^18^ thereby minimizing the risk of misdiagnoses. Only recently has it been shown that the diagnosis established during CRS-R assessment varies with the time of day.^19^ Beyond this, examining circadian processes may also inform about targets for therapeutic interventions such as light stimulation, which has proven successful in individuals with circadian sleep disorders (see reference 20 for a review). Few studies have examined circadian rhythms in DOC patients taking into account variations in hormone secretion^21^ as well as blood pressure and heart rate.^22,23^ Bekinschtein et al.^24^ measured skin temperature during 2 weeks in 5 DOC patients who were in VS/UWS and found that those with traumatic etiology had a circadian temperature rhythm whereas those with anoxic brain damage did not, which they concluded may be due to the extent and severity of the lesion. Matsumoto et al.^25^ recorded core body temperature for 72 hours in 10 elderly DOC patients and found a period length of 24 hours in 7 patients and 6, 12, and 63 hours in the other 3. However, conclusions are difficult to reach from these 2 studies, because sample sizes were small, samples were highly heterogeneous (e.g., included patients with dementia as well as brain injury), or ambient light levels were not controlled for.

The aim of the present study was to investigate circadian temperature rhythms in a larger DOC patient population also covering the whole range of DOC states, that is, VS/UWS, MCS, and MCS_exit_. More specifically, we studied temperature rhythms under a habitual light (HL) condition for 1 week and tested (in a subsample) the potential of bright light stimulation (BLS) to enhance circadian rhythmicity and improve entrainment to a 24-hour zeitgeber.

## METHODS

### Patients.

A total of 20 patients (14 females) were included in the study sample, 8 of whom (3 female) completed both HL and BLS conditions. Two patients were excluded from further analyses. For more detailed information on patients, see table 1, figure e-1, and supplementary material at Neurology.org.

**Table 1:**
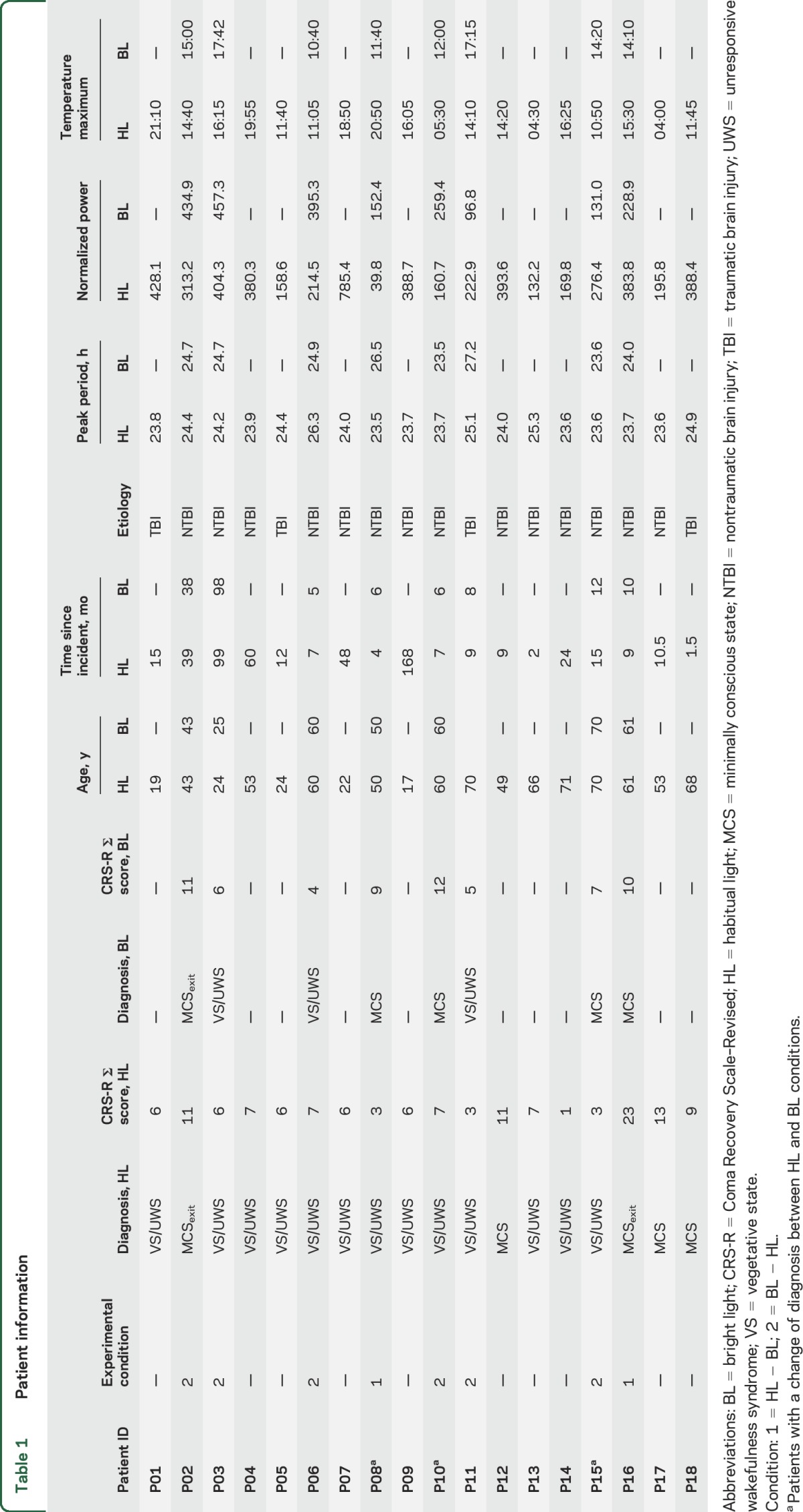
Patient information

### Standard protocol approvals, registrations, and patient consents.

Informed consent was obtained from the patients' legal representatives and approval of the local ethical committee was obtained.

### Experimental protocol.

The study protocol comprised 1 to 2 weeks: 1 week in the HL and for a subsample a second week with BLS. Patients were assessed behaviorally with the CRS-R^18^ by 2 trained experts at the end of each week. For an overview of the CRS-R assessment results, see tables 1, e-1, and e-2. For further details on the experimental design, see the supplementary material.

### Physiologic data collection and analysis.

**Figure 1:**
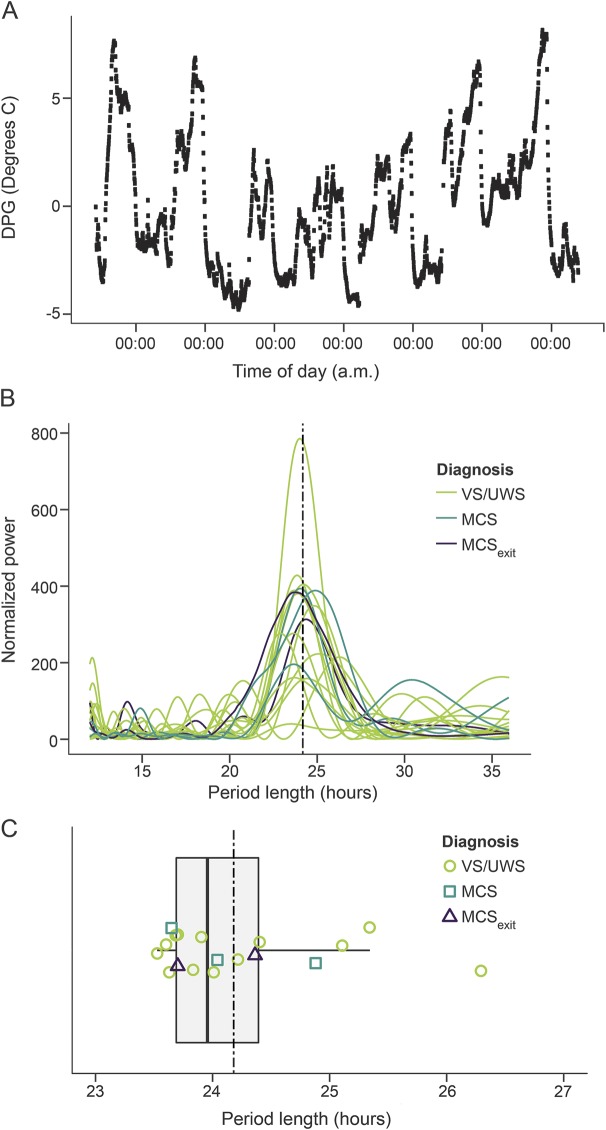
Skin temperature variations, Lomb-Scargle periodogram results, and boxplot of period lengths of circadian rhythms (A) Skin temperature variations. Example data from one patient (P01) show variations in the distal–proximal skin temperature gradient (DPG) across approximately 7 days. (B) Lomb-Scargle periodogram results. This plot shows the results of the Lomb-Scargle periodogram analysis for all participants. The x-axis shows the period length in hours, the y-axis shows the normalized power (arbitrary units). The dashed vertical line indicates 24.18 hours. (C) Boxplot of period lengths of circadian rhythms. The dashed vertical line indicates 24.18 hours, i.e., the supposedly ideal period length reported in well-controlled studies on healthy individuals. The box shows the quartiles, the vertical line in the box represents the median. Whiskers of the boxplot indicate the 1st and 3rd quartile ±1.5 times the interquartile difference. Note that all patients show a circadian temperature rhythm. MCS = minimally conscious state; UWS = unresponsive wakefulness syndrome; VS = vegetative state.

Temperature data were collected using external skin sensors (iButton DS1922L; Maxim Integrated Products, Inc., San Jose, CA). In total, 4 sensors were placed on the patients' skin, 2 in a proximal location relative to the center of the body and 2 in a distal location. For data processing and analysis, we used R version 3.2.5.^26^ Artefacts were removed automatically and, if necessary, data were inspected manually. Following data preprocessing, the distal–proximal skin temperature gradient (DPG) was computed. This gradient has been shown to parallel changes in core body temperature and thus serves as a proxy for it.^27,28^ First, values from proximal and distal sensors were pooled. Subsequently, distal values were subtracted from the proximal ones (see figure 1A for a DPG example). The DPG then served as the input for the computation of the Lomb-Scargle periodogram,^29,30^ a method that can be used to detect rhythms in time series data (see figure 1B for individual periodogram analysis results). We calculated 2 parameters for each patient: (1) the peak period, i.e., the period with the strongest contribution to the variability in the data; and (2) the normalized power. Specifically, we looked for the peak period closest to 24.18 hours (i.e., circadian peak, as the unmasked endogenous period of the human temperature rhythm is 24.18 hours on average).^31^ Besides the peak period, we were also interested in the normalized power, i.e., a goodness of fit measure of the periodicity or strength of a rhythm, corresponding to the circadian peak. Table 1 provides an overview of the peak period and normalized power for each patient and condition. The significance level of the periodogram analyses was set to α = 0.01. In addition, we applied a method previously suggested for the analysis of actimetry data (for methodologic details, see reference 32). We were specifically interested in the interdaily stability (IS) of the rhythm, an index that informs about how well the patients' temperature rhythms were entrained to a 24-hour zeitgeber (i.e., the light–dark cycle). IS should thus mirror both period length as well as normalized power of the circadian peak. For explorative purposes, we also calculated and report the time of occurrence of the temperature maximum for each patient (see figure e-2 for times of occurrence in the HL and figure e-3 for a comparison of HL and bright light conditions). For more details on the physiologic data analysis, see the supplementary material.

### Statistical analyses.

We investigated group differences in period length, normalized power, and interdaily stability (dependent variables) in the HL condition between diagnosis (i.e., VS/UWS, MCS, and MCS_exit_), consciousness (VS/UWS vs MCS/MCS_exit_), as well as the etiology subgroups (traumatic brain injury [TBI] and nontraumatic brain injury [NTBI]) using advanced nonparametric approaches.^33^ Here, we report the analysis of variance (ANOVA) type test with permutation test *p* values (50,000 permutations). We also investigated the relationship between patients' CRS-R scores (total sumscore as well as subscale scores), the deviation of the period length from 24.18 hours, normalized power of the circadian peak in the periodogram, and interdaily stability of the temperature rhythm using the Kendall tau. For all analyses, the significance level was α = 0.05 with *p* values < 0.1 being interpreted as marginally significant. For the comparison of the condition differences in period length, normalized power, and interdaily stability (dependent variables) in the HL and the BLS condition, we used advanced nonparametric methods for repeated measures designs,^34^ for which we report the ANOVA-type statistic. The number of VS/UWS vs MCS/MCS_exit_ diagnoses in each of the conditions was analyzed using the McNemar χ^2^ test. For more details on the statistics, please see the supplementary material.

## RESULTS

### HL condition.

Lomb-Scargle periodogram analyses revealed circadian rhythms in all patients with period lengths ranging from 23.5 to 26.3 hours (median 23.95 hours) (figure 1, B and C). Patients with traumatic etiology (i.e., TBI) exhibited a marginally significantly longer period length than patients with nontraumatic etiology (TBI: mdn = 24.64 hours vs NTBI: mdn = 23.8 hours; *T*_*A*__1,9.75_ = 4.4, *p* = 0.096). For further (nonsignificant) group analyses, see the supplementary material.

**Figure 2:**
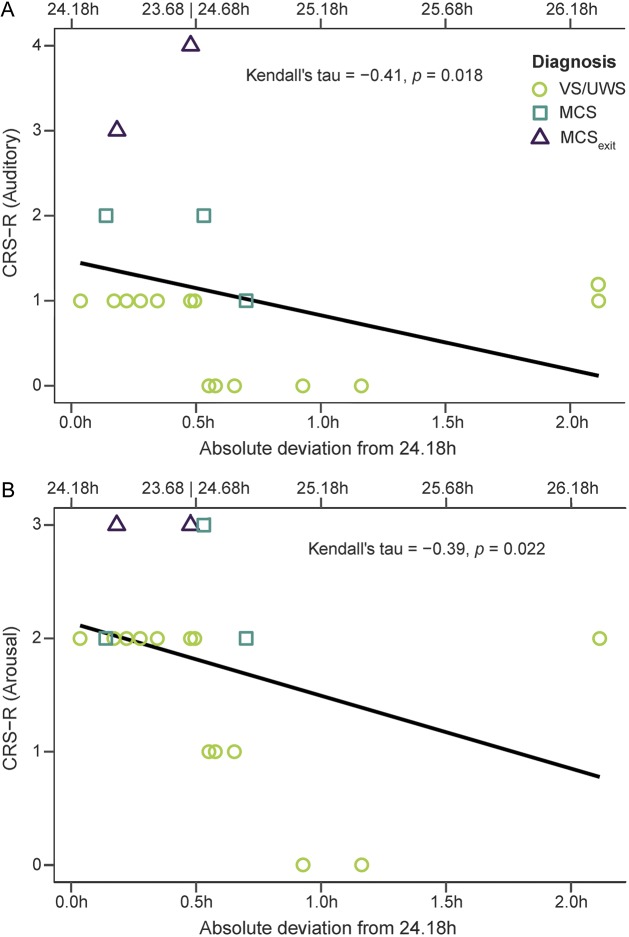
Correlations between Coma Recovery Scale–Revised (CRS-R) and the absolute deviation from 24.18 hours Correlation plots between the CRS-R scores on the (A) auditory and (B) arousal subscales as well as the absolute deviation from the ideal period length of 24.18 hours (see upper x-axis for corresponding period lengths). Note that the less the circadian temperature rhythm deviates, the higher the scores on the CRS-R subscales, i.e., the better the behavioral state of the patient. MCS = minimally conscious state; UWS = unresponsive wakefulness syndrome; VS = vegetative state.

Correlation analyses of the relationship between circadian period length and subscales of the CRS-R indicated that the less patients' rhythms deviated from 24.18 hours, the higher they scored on the auditory (τ = −0.41, *p* = 0.018) and the arousal (τ = −0.39, *p* = 0.022) subscales (figure 2). Correlations with the oromotor/verbal (τ = −0.31, *p* = 0.066) and the communication (τ = −0.30, *p* = 0.079) subscales were marginally significant.

**Figure 3:**
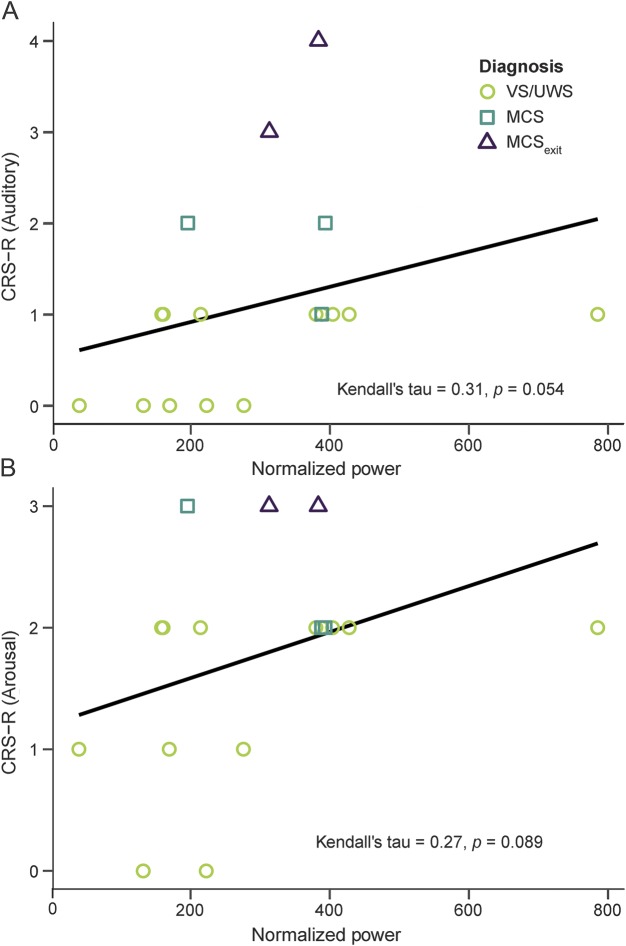
Correlation between Coma Recovery Scale–Revised (CRS-R) and normalized power Correlation plot between the CRS-R scores on the (A) auditory and (B) arousal subscales and normalized power, i.e., the goodness of fit. Note that the more pronounced the circadian rhythm, the higher the scores on the CRS-R subscales, i.e., the better the behavioral state of the patient. MCS = minimally conscious state; UWS = unresponsive wakefulness syndrome; VS = vegetative state.

Correlation analyses between normalized power and CRS-R subscales indicated that higher normalized power was associated with higher scores on the arousal (τ = 0.27, *p* = 0.089) and the auditory (τ = 0.31, *p* = 0.054) subscales with these effects being marginally significant (figure 3).

**Figure 4:**
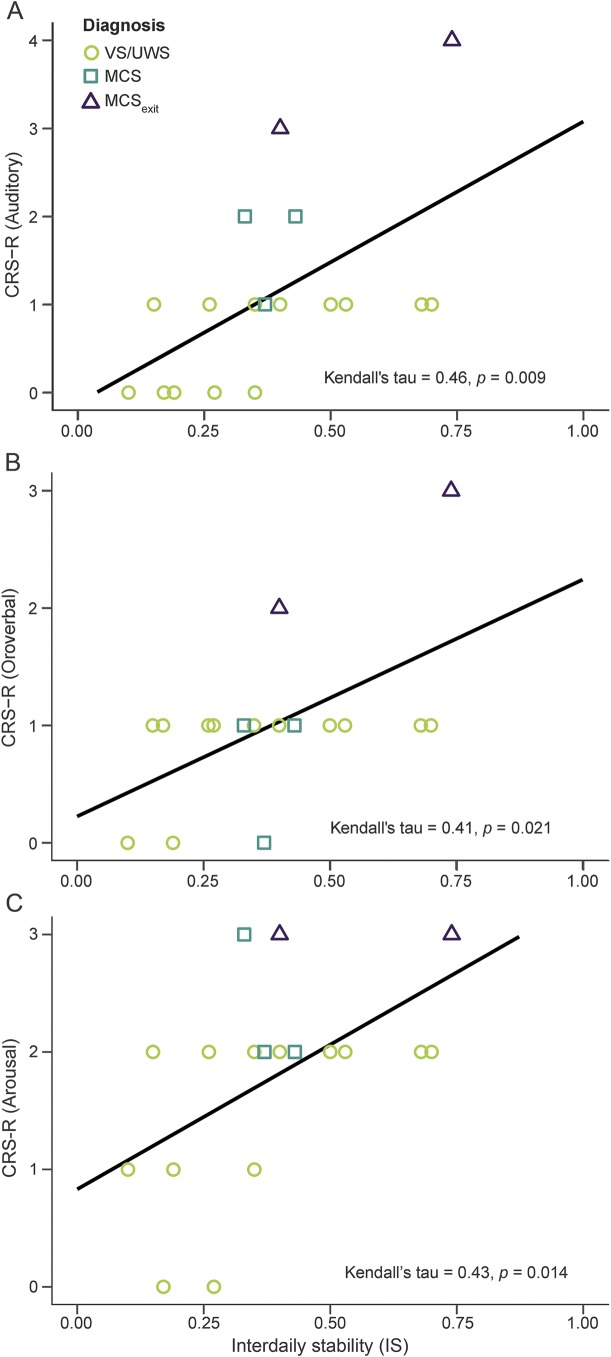
Correlations between Coma Recovery Scale–Revised (CRS-R) and interdaily stability (IS) Correlation plots between the CRS-R scores on the (A) auditory, (B) oromotor, and (C) arousal subscales and interdaily stability (range 0–1; 0 indicates Gaussian noise whereas 1 indicates perfect entrainment to a 24-hour zeitgeber, i.e., the light–dark cycle). Note that the better the temperature rhythm was entrained to a 24-hour zeitgeber, the higher the scores on the CRS-R subscales, i.e., the better the behavioral state of the patient. MCS = minimally conscious state; UWS = unresponsive wakefulness syndrome; VS = vegetative state.

Analyses of correlations between IS and CRS-R values revealed correlations with the auditory (τ = 0.46, p = 0.009), the oromotor/verbal (τ = 0.41, p = 0.021), as well as the arousal (τ = 0.43, p = 0.014) subscales (figure 4). Besides these correlations, analyses yielded marginally significant correlations between IS and the motor subscale (τ = 0.26, p = 0.096), the communication subscale (τ = 0.33, p = 0.056), and the total CRS-R score (τ = 0.25, p = 0.094). As IS should mirror both period length and normalized power, these results confirm preceding analyses.

### BLS condition.

Statistical comparisons between the 2 conditions did not yield significant results (see supplementary material). Three patients (P8, P10, and P15) showed a change of diagnosis from VS/UWS during the HL condition to MCS or MCS_exit_ during BLS. In these 3 patients, the time of occurrence of the temperature maximum shifted from the evening to noon (P8) and from (early) morning to (after)noon (P10 and P15) hours, respectively (table 1 and figure e-3).

## DISCUSSION

We demonstrate in a clinical sample of severely brain injured (DOC) patients that circadian variations in body temperature are related to the behavioral state of the patients. Importantly and in contrast to earlier studies by Bekinschtein et al.^24^ and Matsumoto et al.,^25^ we detected circadian rhythms in all patients irrespective of etiology or diagnosis, which may be due to increased sensitivity of our analysis methods. More precisely, our results indicate that the less the patients' circadian temperature rhythm deviated from healthy rhythmicity, i.e., the better it was entrained to the 24-hour light–dark cycle and the more pronounced the circadian rhythm (i.e., the higher the normalized power of the circadian peak in the periodogram), the better the behavioral repertoire and the state of the patient (as measured with the CRS-R). Generally, our findings are well in line with studies ascribing pathologic significance to disturbances of circadian rhythms in critically ill patients.^14,15^

Although the relationship between the circadian indexes investigated here and the patient's state was also observable on other subscales of the CRS-R, it was especially pronounced for the arousal subscale. Importantly, despite the arousal level not being a diagnostic criterion for VS/UWS or MCS, a patient's arousal level is inherently related to the diagnosis and, thus, other subscales. This is because sufficient cortical arousal or wakefulness promoted by regions in the brainstem such as the ascending reticular activating system is a necessary background condition for consciousness and indeed in simplified descriptions of consciousness it is often conceptualized as the combination of 2 factors, arousal and awareness (for reviews, see references 35 and 36). Functionally, body temperature is thought to be crucially involved in the regulation and stabilization of sleep–wake cycles and thereby also in the stabilization of arousal levels, which are known to fluctuate in DOC patients (for a review, see references 28 and 36). Thus, preserved circadian temperature rhythms may stabilize the integrity of patients' sleep–wake patterns, which in turn would support sustained arousal and eventually attention and (residual) awareness. From a clinical perspective, this renders circadian rhythms promising targets for therapeutic approaches and our findings therefore make a case for treatment aiming at the promotion and stabilization of circadian rhythms.

Generally, ambient light is the key zeitgeber for entraining the biological master clock in the suprachiasmatic nuclei (SCN) of the hypothalamus,^4^ whose output regulates a wide range of processes throughout the entire organism including body temperature. Light exposure has been effectively used for treating circadian rhythm sleep disorders in normal healthy people as well as in clinical populations.^37^ In a pilot study, we therefore also tested the effects of BLS in a subsample of patients. In this protocol, patients received BLS 3 times per day (7 am, 1 pm, and 7 pm) for 1 hour over the course of 1 week. Interestingly, in 3 of these patients who were classified as VS/UWS during the HL condition, classification in the BLS condition changed to MCS/MCS_exit_. As 2 of these patients were assessed under BLS conditions first and only 1 and 3.5 months later under HL conditions, the factor time is unlikely to account for the observed improvements. Thus, these results provide some support for the notion that BLS may have had a beneficial effect on consciousness levels. Statistically, however, no effects of BLS were evident in this first small pilot sample. We suggest that a proof of principle study should be considered before drawing conclusions about the usefulness of BLS. Specifically, the light stimulation protocol introduced here should be tested with a larger sample size allowing for a stratification of the sample according to etiology and severity of disturbance of the circadian rhythm thereby allowing for a differential evaluation of the effects. Such a study may eventually also allow for the evaluation of the potential influence of factors such as sex, age, or accompanying medical conditions on circadian temperature rhythms.

Interestingly, in the 3 patients who showed behavioral improvement with BLS, the temperature maximum, which has previously been found to occur at about the same time as the circadian peak in alertness and performance in healthy individuals (see reference 7 for a review), was shifted. Specifically, it occurred closer to the times when the temperature maximum would be expected in healthy individuals^6^ and closer to the times of the CRS-R assessments in these cases, thereby underlining the potential clinical usefulness of BLS. Even more importantly, we can speculate that a prespecified temperature rhythm could be useful in guiding the time of assessment of patients, thereby decreasing the risk of misdiagnoses, and we propose that it may be advantageous if assessments took place around the time of occurrence of the temperature maximum. Future studies should specifically test this hypothesis, i.e., that behavioral performance improves the closer to the temperature maximum the assessment takes place. Beyond this, the variability in the times when patients' body temperature was maximal (figure e-2) may further underline the significance of light/dark cycles on DOC patients. While light levels were generally low (<500 lux, figure e-1), patients varied in the amount of time their eyes were closed and ambient light levels were modulated by, e.g., weather conditions. Although very low light levels (1.5 lux) have been shown to be sufficient to entrain circadian rhythms in healthy individuals,^38^ this may be different in clinical populations.

A possible limitation of the present study is that no (magnetic resonance) imaging data were available to evaluate the extent of brain injury and potential damage to the hypothalamus and the SCN in particular. However, especially the circadian temperature rhythm has been proposed to be robust. Rodent studies have shown that even damage to the SCN that only spares a small number of cells does not eliminate circadian temperature rhythms, although it may alter them.^39^ Future studies should extend the findings presented here to other body rhythms and examine the coupling among different oscillators (e.g., hormones, rest-activity cycles, temperature). Moreover, they should investigate the relationship of variations in peripheral rhythms such as body temperature to variations in well-established measures of central brain activity that are known to differentiate reliably between different states of consciousness in healthy individuals as well as DOC patients independently from behavioral signs of consciousness such as the so-called perturbational complexity index,^40^ which can be derived from studies combining transcranial magnetic stimulation and EEG. Recent research by Ly et al.^11^ for example suggests that body temperature variations could actually be causally involved in the regulation of cortical excitability.

Our results show that the integrity of circadian temperature rhythms is related to the behavioral repertoire and therefore the state of a patient as measured by the CRS-R in a sample of severely brain-injured individuals. This relationship is especially pronounced for arousal levels, a precondition for consciousness, thereby also suggesting that patients' circadian rhythms may represent an interesting therapeutic target. BLS, which is easy to apply at bedside and cost-efficient, may depict one such therapeutic approach.

## Supplementary Material

Data Supplement

## GLOSSARY

ANOVA: analysis of variance
BLS: bright light stimulation
CRS-R: Coma Recovery Scale–Revised
DOC: disorder of consciousness
DPG: distal–proximal skin temperature gradient
HL: habitual light
IS: interdaily stability
MCS: minimally conscious state
NTBI: nontraumatic brain injury
SCN: suprachiasmatic nuclei
TBI: traumatic brain injury
UWS: unresponsive wakefulness syndrome
VS: vegetative state

## References

[1] Storch KF, Lipan O, Leykin I, et al. Extensive and divergent circadian gene expression in liver and heart. Nature 2002;417:78–83. 11967526 10.1038/nature744

[2] Dijk DJ, Duffy JF, Czeisler CA. Circadian and sleep/wake dependent aspects of subjective alertness and cognitive performance. J Sleep Res 1992;1:112–117. 10607036 10.1111/j.1365-2869.1992.tb00021.x

[3] Santhi N, Lazar AS, McCabe PJ, Lo JC, Groeger JA, Dijk DJ. Sex differences in the circadian regulation of sleep and waking cognition in humans. Proc Natl Acad Sci USA 2016;113:E2730–E2739. 27091961 10.1073/pnas.1521637113 PMC4868418

[4] Arendt J, Broadway J. Light and melatonin as zeitgebers in man. Chronobiol Int 1987;4:273–282. 3334221 10.3109/07420528709078534

[5] Inouye SI, Kawamura H. Persistence of circadian rhythmicity in a mammalian hypothalamic “island” containing the suprachiasmatic nucleus. Proc Natl Acad Sci USA 1979;76:5962–5966. 293695 10.1073/pnas.76.11.5962 PMC411773

[6] Wyatt JK, Cecco ARD, Czeisler CA, Dijk DJ. Circadian temperature and melatonin rhythms, sleep, and neurobehavioral function in humans living on a 20-h day. Am J Physiol 1999;277:R1152–R1163. 10516257 10.1152/ajpregu.1999.277.4.r1152

[7] Schmidt C, Collette F, Cajochen C, Peigneux P. A time to think: circadian rhythms in human cognition. Cogn Neuropsychol 2007;24:755–789. 18066734 10.1080/02643290701754158

[8] Tononi G. An information integration theory of consciousness. BMC Neurosci 2004;5:42. 15522121 10.1186/1471-2202-5-42 PMC543470

[9] Tononi G. Consciousness as integrated information: a provisional manifesto. Biol Bull 2008;215:216–242. 19098144 10.2307/25470707

[10] Dehaene S, Changeux JP, Naccache L, Sackur J, Sergent C. Conscious, preconscious, and subliminal processing: a testable taxonomy. Trends Cogn Sci 2006;10:204–211. 16603406 10.1016/j.tics.2006.03.007

[11] Ly JQM, Gaggioni G, Chellappa SL, et al. Circadian regulation of human cortical excitability. Nat Commun 2016;7:11828. 27339884 10.1038/ncomms11828 PMC4931032

[12] Wright JE, Vogel J, Sampson J, Knapik J, Patton J, Daniels W. Effects of travel across time zones (jet-lag) on exercise capacity and performance. Aviat Space Environ Med 1983;54:132–137. 6838449

[13] Santhi N, Horowitz TS, Duffy JF, Czeisler CA. Acute sleep deprivation and circadian misalignment associated with transition onto the first night of work impairs visual selective attention. PLoS One 2007;2:e1233. 18043740 10.1371/journal.pone.0001233 PMC2077929

[14] Mundigler G, Delle-Karth G, Koreny M, et al. Impaired circadian rhythm of melatonin secretion in sedated critically ill patients with severe sepsis. Crit Care Med 2002;30:536–540. 11990911 10.1097/00003246-200203000-00007

[15] Gehlbach BK, Chapotot F, Leproult R, et al. Temporal disorganization of circadian rhythmicity and sleep-wake regulation in mechanically ventilated patients receiving continuous intravenous sedation. Sleep 2012;35:1105–1114. 22851806 10.5665/sleep.1998 PMC3397814

[16] Giacino JT, Malone R. The vegetative and minimally conscious states. Handbook Clin Neurol 2008;90:99–111. 10.1016/S0072-9752(07)01706-X 18631819

[17] Bruno MA, Vanhaudenhuyse A, Thibaut A, Moonen G, Laureys S. From unresponsive wakefulness to minimally conscious PLUS and functional locked-in syndromes: recent advances in our understanding of disorders of consciousness. J Neurol 2011;258:1373–1384. 21674197 10.1007/s00415-011-6114-x

[18] Kalmar K, Giacino JT. The JFK coma recovery scale: revised. Neuropsychol Rehabil 2005;15:454–460. 16350986 10.1080/09602010443000425

[19] Cortese M, Riganello F, Arcuri F, et al. Coma recovery scale-r: variability in the disorder of consciousness. BMC Neurol 2015;15:186. 26450569 10.1186/s12883-015-0455-5 PMC4599033

[20] LeGates TA, Fernandez DC, Hattar S. Light as a central modulator of circadian rhythms, sleep and affect. Nat Rev Neurosci 2014;15:443–454. 24917305 10.1038/nrn3743 PMC4254760

[21] Guaraldi P, Sancisi E, La Morgia C, et al. Nocturnal melatonin regulation in post-traumatic vegetative state: a possible role for melatonin supplementation? Chronobiol Int 2014;31:741–745. 24679225 10.3109/07420528.2014.901972

[22] Fukudome Y, Abe I, Saku Y, et al. Circadian blood pressure in patients in a persistent vegetative state. Am J Physiol 1996;39:R1109–R1114. 10.1152/ajpregu.1996.270.5.R1109 8928913

[23] Pattoneri P, Tirabassi G, Pelà G, Astorri E, Mazzucchi A, Borghetti A. Circadian blood pressure and heart rate changes in patients in a persistent vegetative state after traumatic brain injury. J Clin Hypertens 2005;7:734–739. 10.1111/j.1524-6175.2005.04780.x PMC8109359 16330896

[24] Bekinschtein TA, Golombek DA, Simonetta SH, Coleman MR, Manes FF. Circadian rhythms in the vegetative state. Brain Inj 2009;23:915–919. 20100128 10.1080/02699050903283197

[25] Matsumoto M, Sugama J, Okuwa M, Dai M, Matsuo J, Sanada H. Non-invasive monitoring of core body temperature rhythms over 72h in 10 bedridden elderly patients with disorders of consciousness in a Japanese hospital: a pilot study. Arch Gerontol Geriatr 2013;57:428–432. 23866791 10.1016/j.archger.2013.05.009

[26] R Core Team. R: A Language and Environment for Statistical Computing. Vienna: R Foundation for Statistical Computing; 2015.

[27] Hasselberg MJ, McMahon J, Parker K. The validity, reliability, and utility of the iButton (R) for measurement of body temperature circadian rhythms in sleep/wake research. Sleep Med 2013;14:5–11. 21470909 10.1016/j.sleep.2010.12.011

[28] Kräuchi K, Cajochen C, Werth E, Wirz-Justice A. Physiology: warm feet promote the rapid onset of sleep. Nature 1999;401:36–37. 10485703 10.1038/43366

[29] Lomb NR. Least-squares frequency analysis of unequally spaced data. Astrophysics Space Sci 1976;39:447–462.

[30] Scargle JD. Studies in astronomical time series analysis: II: statistical aspects of spectral analysis of unevenly spaced data. Astrophys J 1982;263:835–853.

[31] Czeisler CA, Duffy JF, Shanahan TL, et al. Stability, precision, and near-24-hour period of the human circadian pacemaker. Science 1999;284:2177–2181. 10381883 10.1126/science.284.5423.2177

[32] Blume C, Santhi N, Schabus M. “nparACT” package for R: a free software tool for the non-parametric analysis of actigraphy data. MethodsX 2016;3:430–435. 27294030 10.1016/j.mex.2016.05.006 PMC4890079

[33] Burchett WW, Ellis AR, Harrar SW, Bathke AC. Nonparametric inference for multivariate data: the R package npmv. J Stat Softw 2017;76:18.

[34] Noguchi K, Gel YR, Brunner E, Konietschke F. nparLD: an R software package for the nonparametric analysis of longitudinal data in factorial experiments. J Stat Softw 2012;50:1–23.25317082

[35] Koch C, Massimini M, Boly M, Tononi G. Neural correlates of consciousness: progress and problems. Nat Rev Neurosci 2016;17:307–321. 27094080 10.1038/nrn.2016.22

[36] Blume C, del Giudice R, Wislowska M, Lechinger J, Schabus M. Across the consciousness continuum: from unresponsive wakefulness to sleep. Front Hum Neurosci 2015;9:105. 25805982 10.3389/fnhum.2015.00105 PMC4354375

[37] Ancoli-Israel S, Gehrman P, Martin JL, et al. Increased light exposure consolidates sleep and strengthens circadian rhythms in severe Alzheimer's disease patients. Behav Sleep Med 2003;1:22–36. 15600135 10.1207/S15402010BSM0101_4

[38] Wright KP, Hughes RJ, Kronauer RE, Dijk DJ, Czeisler CA. Intrinsic near-24-h pacemaker period determines limits of circadian entrainment to a weak synchronizer in humans. Proc Natl Acad Sci USA 2001;98:14027–14032. 11717461 10.1073/pnas.201530198 PMC61161

[39] Refinetti R, Menaker M. The circadian rhythm of body temperature. Physiol Behav 1992;51:613–637. 1523238 10.1016/0031-9384(92)90188-8

[40] Casali AG, Gosseries O, Rosanova M, et al. A theoretically based index of consciousness independent of sensory processing and behavior. Sci Transl Med 2013;5:198ra105. 10.1126/scitranslmed.3006294 23946194

