# Supplementary material for: Significance of circadian rhythms in severely brain-injured patients: A clue to consciousness?
Source: Neurology. 2017 May 16;88(20):1933–41. doi: 10.1212/WNL.0000000000003942 (PMC5444311; doi:10.1212/WNL.0000000000003942)
Supplement: Data Supplement [file supp_88_20_1933__index.html]

Significance of circadian rhythms in severely brain-injured patients — Data Supplement 

# Significance of circadian rhythms in severely brain-injured patients

## Data Supplement

**Neurology® data supplements are not copyedited before publication. Published editorials and translations have been copyedited.  
 © 2017 American Academy of Neurology.  
  
 Files in this Data Supplement:**

- Data Supplement - Microsoft Word file
