# Supplementary material for: Significance of circadian rhythms in severely brain-injured patients: A clue to consciousness?
Source: Neurology. 2017 May 16;88(20):1933–41. doi: 10.1212/WNL.0000000000003942 (PMC5444311; doi:10.1212/WNL.0000000000003942)
Supplement: Data Supplement [file supp_WNL.0000000000003942_CircadianTemperature_Supplementary_Material_09_02_16__3_Neurology_clean.docx]

**A clue to consciousness? Significance of circadian rhythms in severely brain-injured patients**

SUPPLEMENTARY MATERIAL

Dr Christine Blume^1,2^, Dr Julia Lechinger^1,2^, Dr Nayantara Santhi^3^, Dr Renata del Giudice^1,2^, MSc Maria-Teresa Gnjezda^1^, Gerald Pichler M.D.^5^, Monika Scarpatetti M.D.^5^, Johann Donis M.D.^6^, Gabriele Michitsch M.D.^6^, Prof. Dr. Manuel Schabus^1,2^

^1^ University of Salzburg, Department of Psychology, Laboratory for Sleep, Cognition and Consciousness Research, Hellbrunner Strasse 34, 5020 Salzburg

^2^ University of Salzburg, Centre for Cognitive Neuroscience Salzburg (CCNS), Hellbrunner Strasse 34, 5020 Salzburg

^3^ Surrey Sleep Research Centre, Faculty of Health and Medical Sciences, University of Surrey, Egerton Road, Guildford GU2 7XP, United Kingdom

^5^ Geriatric Health Centres of the City of Graz, Albert Schweitzer Clinic, Apallic Care Unit, Albert-Schweitzer-Gasse 36, 8020 Graz, Austria

^6^ Pflegewohnhaus Donaustadt Wien, Neurologische Abteilung mit Wachkomabetreuung, Langobardenstrasse 122a, 1220 Vienna, Austria

**Corresponding Author:**

Dr. Christine Blume

University of Salzburg

Centre for Cognitive Neuroscience (CCNS)

Laboratory for “Sleep, Cognition and Consciousness Research”

Hellbrunner Str. 34

A-5020 Salzburg

Email: [christine.blume@sbg.ac.at](mailto:christine.blume@sbg.ac.at)

T: +43(0)662 8044-5146

**Methods and Materials**

***Patients***

A total of *N* = 20 patients were included in the study sample, eight of whom completed both conditions, i.e. habitual and bright light. Patients were recruited from long-term care facilities in Austria and did not suffer from dysautonomia or acute illnesses such as infections with fever, which could have affected the measurements. Also, they were free from medication that is known to affect circadian rhythmicity such as melatonin. All patients in the cooperating centres in Austria that fulfilled the criteria and for whom informed consent was obtained were included in the study. As magnetic resonance imaging (MRI) data were not accessible the extent of brain damage, which may have involved hypothalamic damage, is unclear. All patients presented with alternating periods of open and closed eyes, which could represent periods of wakefulness and sleep, respectively. Data from two patients were excluded from further analyses, one because the sampling rate had accidentally been set to 600s (i.e. one sampling point every ten minutes) and the other one because the patient had inadvertently been included in the study (he was not MCS_exit_ but rather suffered from mild cognitive impairment). Of the 18 patients, (14 females) 13 were in VS/UWS, three in MCS and two in MCS_exit_ during the HL condition (for details see Table 1 and Table e-1). Eight patients (three females) additionally underwent bright light stimulation (BLS), three of whom were in VS/UWS, four in MCS and one in MCS_exit_. For these patients, the order of the conditions was randomised. Informed consent was obtained from the patients’ legal representatives and approval of the local ethical committee had been obtained.

***Experimental design***

The study protocol comprised one to two weeks, that is one week in the habitual light (HL) condition and for a subsample another week during which patients received bright light (BL) stimulation. During both weeks the light in the patients’ rooms was switched on at 7am and off at 9pm to keep a constant light-dark rhythm. Although this rhythm slightly deviates from the normal 2:1 ratio of wakefulness and sleep that is usually present in healthy individuals (Cohen, Wang, Wyatt et al., 2010), it was easiest to implement in the hospital environment and closest to the light-dark cycle there. Patients were moved every 3-4 hours during day and night and continued to live in their habitual hospital environment (including therapies, visits, nursing etc.). A dim light (< 10 lux at patients’ eye level) was switched on when nurses entered the patient’s room during the night (usually twice per night for approx. 5-10 minutes) with patients being in complete darkness for the rest of the night. Blinds were closed during the lights off period to ensure low light levels even when the sun rose earlier than 7am during the summer months. All patients had a stomach tube via which they received nutrition on a more or less constant level with breaks being e.g. due nurses having to replace empty nutrition bags.

*Habitual Light Condition*

During the HL week, ambient light levels were kept below 500 lux at eye level at all times during the day, i.e. between 7am and 9pm. The architecture generally prevented light levels from exceeding 500 lux in the patients’ rooms. Moreover, light levels were tracked by continuous recordings of ambient light levels with actigraphs (wGT3X-BT Monitor, ActiGraph LLC., Pensacola, USA) that were placed at the bedhead in a way their orientation matched the patients’ perspective as closely as possible. Spot-checks with a digital luxmeter (Dr. Meter, Digital Illuminance/Light Meter LX1330B) confirmed that light levels did not exceed 500 lux at eye level. This was important as the actigraphs are characterised by reduced sensitivity especially at low light levels and it cannot be excluded that they were for example inadvertently and unnoticedly covered by bed sheets between regular checks by the research team.


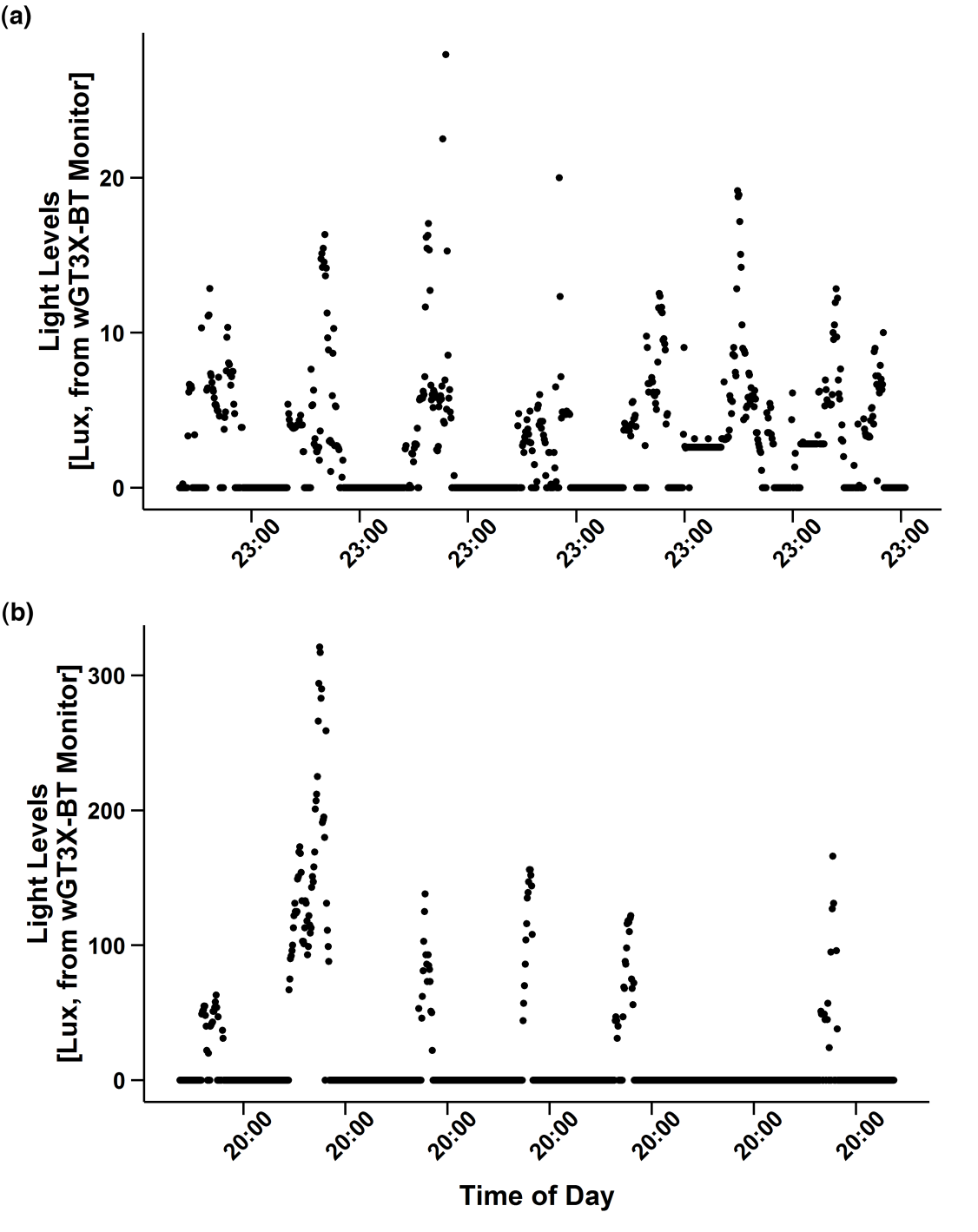


**Figure e-1: (a) Ambient Light Levels measured with the wGT3X-BT Device averaged across Recordings in the Habitual Light Condition.** The plot shows maximal light levels in 10min bins averaged across all recordings in the habitual light condition. **(b) Ambient Light Levels from one Recording.** The plot exemplarily shows maximal light levels in 10min bins in the habitual light condition for one recording (at the facility with the brightest rooms). Please note that light levels below approx. 50 lux cannot be recorded reliably with the wGT3X-BT device, wherefore low light levels appear as zeroes. This also accounts for the, on average, very low lux levels (see upper panel).

Patients were not exposed to sunlight or wore dark sunglasses covering the whole eye when having to leave the building e.g. for medical reasons.

*Bright Light Condition*

During the BL week, patients were stimulated with bright and blue-enriched light (≈ 2000 lux at eye level) three times a day for one hour. BL stimulation took place from 7 to 8am, from 1 to 2pm and from 7 to 8pm. Luminette® light goggles were used for the stimulation.

***Behavioural assessment***

Patients were assessed behaviourally with the Coma-Recovery-Scale Revised (CRS-R, Kalmar & Giacino, 2005) by two trained experts on days six and seven of each study week (i.e. HL and, if applicable, BL). The CRS-R comprises 23 items that are grouped into six subscales reflecting auditory, visual, motor, oromotor, communication and arousal functions. Items of each subscale are arranged hierarchically with the lowest item representing reflexive activity indicating brain stem functioning and the highest item representing cognitively mediated behaviours involving cortical processes. The subscales that are particularly relevant to this publication as results revealed significant correlations with them are the auditory, the oromotor/verbal and the arousal subscales. The auditory subscale consists of five items: 0 = “No Response”; 1 = “Auditory Startle”; 2 = “Localization to Sound”, 3 = “Reproducible Movement to Command”, 4 = “Consistent Movement to Command”. The oromotor/verbal subscale comprises four items: 0 = “No Response”, 1 = “Oral Reflexive Movement”, 2 = “Vocalization/Oral Movement”, 3 = “Intelligible Verbalisation” as does the arousal subscale: 0 = “Unarousable”; 1 = “Eye Opening with Stimulation”; 2 = “Eye Opening w/o Stimulation”, 3 = “Attention”. In the HL condition, patients were tested twice, once in the morning on day six and once in the afternoon on day seven. In the BL condition, patients were tested three times, once in the morning of day six and additionally before and directly after the BL stimulation in the afternoon of day seven unless exhaustion precluded the third assessment (for the CRS-R scores before and after BL stimulation please see table e-2). For further analyses, we used the scores obtained during the assessment with the best diagnosis or highest sum score as this is thought to best represent the true state of the patient for each condition. For an overview of the CRS-R assessment results please see Table 1 and Table e-1 for detailed results.

***Physiological assessment***

Temperature data were collected with a sampling rate of 1/300 Hz, i.e. one sample every five minutes, using external skin sensors (iButton DS1922L; Maxim Integrated Products, Inc., San Jose, CA, USA). These sensors have previously been shown to be useful for circadian rhythm research (Hasselberg, McMahon, & Parker, 2013; van Marken Lichtenbelt, Daanen, Wouters et al., 2006). In total, four sensors were placed on the patients’ skin in an infraclavicular position about 7-10 cm left and right of the sternum and thus in a proximal position relative to the centre of the body. For one patient, sensors were placed medially from the anterior axillary line to prevent repeated removal. Two other sensors were placed in a distal position on the insides of the lower legs just above the ankles (cf. Kräuchi, Cajochen, Werth, & Wirz-Justice, 1999; Kräuchi, Cajochen, Werth, & Wirz-Justice, 2000). All sensors were affixed using adhesive medical tape and the skin was shaved beforehand if necessary. Additionally a sensor was fixed to a bedside table in the patient’s room to record the ambient room temperature.

***Physiological data analysis***

For data processing and analysis we used R version 3.2.5 (R Core Team, 2015). First, data from each sensor were processed individually. Artefacts, which resulted from the sensors being taken off e.g. for showering, were removed. To this end, all values, which fell outside 2.5 times the interquartile difference from the temperature median of each sensor, were excluded from further analyses. For one patient, who repeatedly took off the infraclavicular sensors during one day, we performed a visual data inspection that was accompanied by manual artefact exclusion. Following this artefact rejection protocol, we included 1865.5 (median) data points (corresponding to approx. 6.5 days) for the HL condition and 1892 (median) data points (corresponding to approx. 6.4 days) for the BL condition.

Following data pre-processing, the proximal-distal skin temperature gradient (DPG) was computed. This gradient has been shown to parallel changes in core body temperature and thus serves as a proxy for it (e.g. Hasselberg, McMahon, & Parker, 2013; Kräuchi, Cajochen, Werth, & Wirz-Justice, 1999), and also circumvents medical and ethical problems related to measuring core body temperature rectally. More specifically, core body temperature is regulated by two mechanisms, (i) core body temperature itself and (ii) heat loss via vasodilatation and constriction in the periphery (Kräuchi, 2002; Aschoff & Wever, 1958). The interplay between these two contributing factors is mirrored by variations in the DPG. For the calculation of the DPG, values from the two proximal and the two distal sensors were first pooled. Subsequently, the distal values were subtracted from the proximal ones in a pointwise fashion yielding the proximal-distal DPG (see Fig. 1a for an example). Additionally, as recommended by Van Dongen, Olofsen, Van Hartevelt, and Kruyt (1999) before the application of periodogram analyses (see next section), we subtracted the grand average from the gradient in a pointwise manner to account for the “offset”. In two subjects only one value from each site could be obtained, once due to a technical error and once because the second sensor got lost.

*Lomb-Scargle Periodogram Analysis*

The DPG served as the input for the computation of the Lomb-Scargle periodogram (Lomb, 1976; Scargle, 1982). The Lomb-Scargle periodogram is a least squares spectral analysis (LSSA) method that can be used to detect rhythms in time series data. The maximum in the periodogram occurs where the sum of squares of the fit of a sinusoid to the data is minimal, that is where the normalised power is maximal (see Fig. 1b for individual periodogram analysis results).

This method has been shown to be especially suitable for the detection of rhythms in noisy data and levels of significance for each period length in the periodogram can easily be computed. An added advantage is that the Lomb-Scargle periodogram can handle unequally sampled data, thus allowing for artefact-rejection making it useful for the analysis of human data (for a comprehensive discussion of the advantages see Ruf, 1999). For the calculation of the periodogram, we used the “lomb” package available for R (Ruf, 1999) to calculate two parameters for each patient, namely, (i) the peak period and (ii) the normalised power. The peak period is the period length at which the normalised power in the periodogram reaches a maximum, i.e. the period with the strongest contribution to the variability in the temperature data. Specifically, we looked for significant peaks in the periodogram between 24 ± 12 hours (i.e. lower limit at 12 hours, upper limit at 36 hours) to make sure we could catch even rhythmicity that substantially deviated from 24 hours. This approach usually yielded several significant peaks, suggesting that oscillations of different period length contributed to the data to a varying but significant extent. As previous studies have determined the unmasked endogenous period of the human temperature rhythm to be 24.18 hours on average (Czeisler, Duffy, Shanahan et al., 1999), we then looked for the peak closest to 24.18 hours (i.e. “circadian peak”) and declared the corresponding period length as the period length of the patient’s circadian rhythm. In all but one dataset from the HL and two from the BL condition, the global peak was equivalent to the peak closest to 24.18 hours.

Besides the peak period, we were also interested in the normalised power corresponding to the “circadian peak”. As this is a goodness of fit measure of the periodicity, it can be seen as an index for the strength of a rhythm. Table 1 provides an overview of the peak period as well as the normalised power for each patient and recording. The oversampling factor was chosen to be 100 to obtain a fine scanning of frequencies and the significance level was set to α = 0.01.

*Analysis of Interdaily Stability (IS)*

In addition to the periodogram analyses, we also applied a previously suggested method for the analysis of actimetry data implemented in the R package “nparACT” (for methodological details see Blume, Santhi, & Schabus, 2016). Here, we were specifically interested in the *Interdaily Stability* (IS) of the rhythm, i.e. how well the patients’ temperature rhythms were entrained to a 24h zeitgeber (i.e. the light-dark cycle) as indexed by values ranging between 0 for Gaussian noise and 1 for perfect IS. *Interdaily Stability* should thus mirror and integrate both period length as well as normalised power of the “circadian peak”. As this method does not allow for missing values in the time series, missing values were imputed by replacing the first half of missing values with the last valid value and the second half of the missing values with the first valid value following the missing values. When there was only one missing data point it was replaced by the mean of the last and the first valid value. Generally, if missing values spanned more than six hours we removed the data from this whole day carefully weighing loss of data and imputation in this case. This affected the datasets from two patients. Overall, we imputed a median of zero values (range 0-36) in the HL and a median of one value (range 0-26) in the BL condition. Furthermore, as our data contained negative values which could have affected the results of this approach, we linearly transformed data to remove negative values in the data. As this transformation essentially preserved the rhythmicity in the data, results were unaffected.

*Analysis of the temperature maximum*

For explorative purposes, we also calculated and report the occurrence of the temperature maximum for each patient. To this end, we first computed the mean of each sampling point across 24h (i.e. all available values obtained at 3:00pm, at 3:05pm, at 3:10pm etc. were averaged yielding a total of 144 data points) from the DPG data. Next, we averaged data points across consecutive 30min periods (i.e. from 3:00pm to 3:30pm, from 3:05pm to 3:35pm, etc.) to obtain a more reliable estimate of low-frequency changes in body temperature. Finally, we looked for the maximum among all 30min averages and noted the time of occurrence of the temperature maximum.

***Statistical Analyses***

For statistical details on the Lomb-Scargle periodogram analyses please see section “Data Analysis”. We investigated group differences in period length, normalised power and interdaily stability (dependent variables) in the HL condition between DIAGNOSIS (i.e. VS/UWS, MCS and MCS_exit_), CONSCIOUSNESS (VS/UWS vs. MCS/ MCS_exit_) as well as the AETIOLOGY subgroups (traumatic brain injury [TBI] and non-traumatic brain injury [NTBI]) using advanced non-parametric approaches as implemented in the “npmv” package (see Burchett, Ellis, Harrar, & Bathke, 2017 for methodological and mathematical details). Here, we report the ANOVA type test with permutation test *p*-values (50000 permutations). Beyond group analyses, we also calculated Kendall’s tau coefficient as implemented in the “Kendall” package in R (McLeod, 2011) to investigate the relationship between patients’ CRS-R scores (total sum score as well as auditory, visual, motor, communication, oromotor/verbal and arousal subscale scores), the (i) deviation of the supposedly “ideal” period length of 24.18h (Czeisler, Duffy, Shanahan et al., 1999), (ii) normalised power of the circadian peak in the periodogram as well as (iii) interdaily stability of the temperature rhythm. For all analyses, the significance level was α = 0.05 with *p*-values < .1 being interpreted as marginally significant. For correlation analyses we report one-sided *p*-values as a priori hypotheses about their directionality existed. For the comparison of the condition differences in period length, normalised power and interdaily stability (dependent variables) in the HL and the BL condition, we used the non-parametric methods for repeated measures designs implemented in the “nparLD” package (see Noguchi, Gel, Brunner, & Konietschke, 2012 for methodological and mathematical details) for which we report the ANOVA-type statistic. The number of VS/UWS vs. MCS/ MCS_exit_ diagnoses in each of the conditions was analysed using Mc Nemar’s χ^2^ test with continuity correction for small samples.

**Results**

***Habitual Light Condition***

Results from the non-parametric ANOVA type test did not indicate differences in period length between the DIAGNOSIS subgroups (cf. Fig. 1c), i.e. no differences in period length could be observed between VS/UWS, MCS and MCS_exit_ patients (VS/UWS: *mdn* = 23.90h vs. MCS: *mdn* = 24.04h vs. MCS_exit_: *mdn* = 24.03h). Also no differences between unconscious (VS/UWS) and conscious (MCS and MCS_exit_) patients were evident (VS/UWS: *mdn* = 23.90h vs. MCS_(exit)_: *mdn* = 24.04h).

Results of the non-parametric ANOVA type analyses indicated that there were no differences in normalised power or IS between the DIAGNOSIS, CONSCIOUSNESS (VS/UWS vs. MCS/ MCS_exit_ and the AETIOLOGY subgroups.

***Bright Light Condition***

ANOVA type analyses did not yield any significant differences between HL and BL condition regarding period length (*ATS*_1_ = 1.13, *p* = .29), normalised power (*ATS*_1_ = 0.77, *p* = .78) or interdaily stability (*ATS*_1_ = 0.55, *p* = .46). The statistical comparison of the number of VS/UWS vs. MCS/ MCS_exit_ diagnoses between the HL and BL condition did not yield differences (Mc Nemar’s *χ^2^*_1_ = 1.3, *p* = .25).

**Figures**

***Figure e-2***


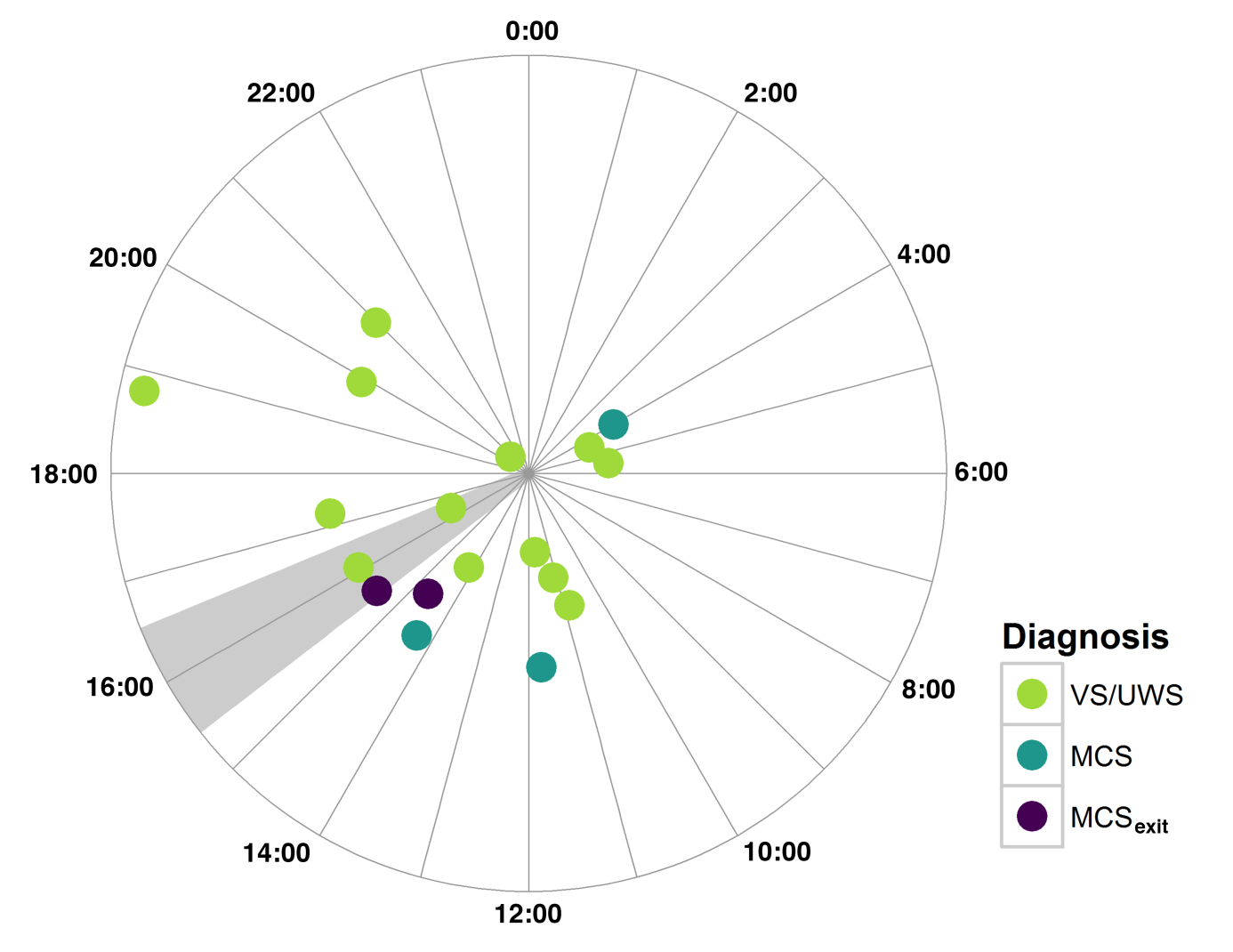


**Figure e-2: Times of Occurrence of the Temperature Maximum in the Habitual Light Condition.** Each dot represents the time of occurrence of one patient’s temperature maximum. The distance of each dot from the centre of the plot indicates the normalised power of the circadian peak, i.e. the strength of the circadian rhythm. The right half of the plot represents the time from midnight until noon, the left half the time from noon until midnight. In healthy, day-active individuals the temperature maximum would be expected at about 16:00 hours, the grey area indicates the time period between 15:30 and 16:30 hours.

***Figure e-3***

**
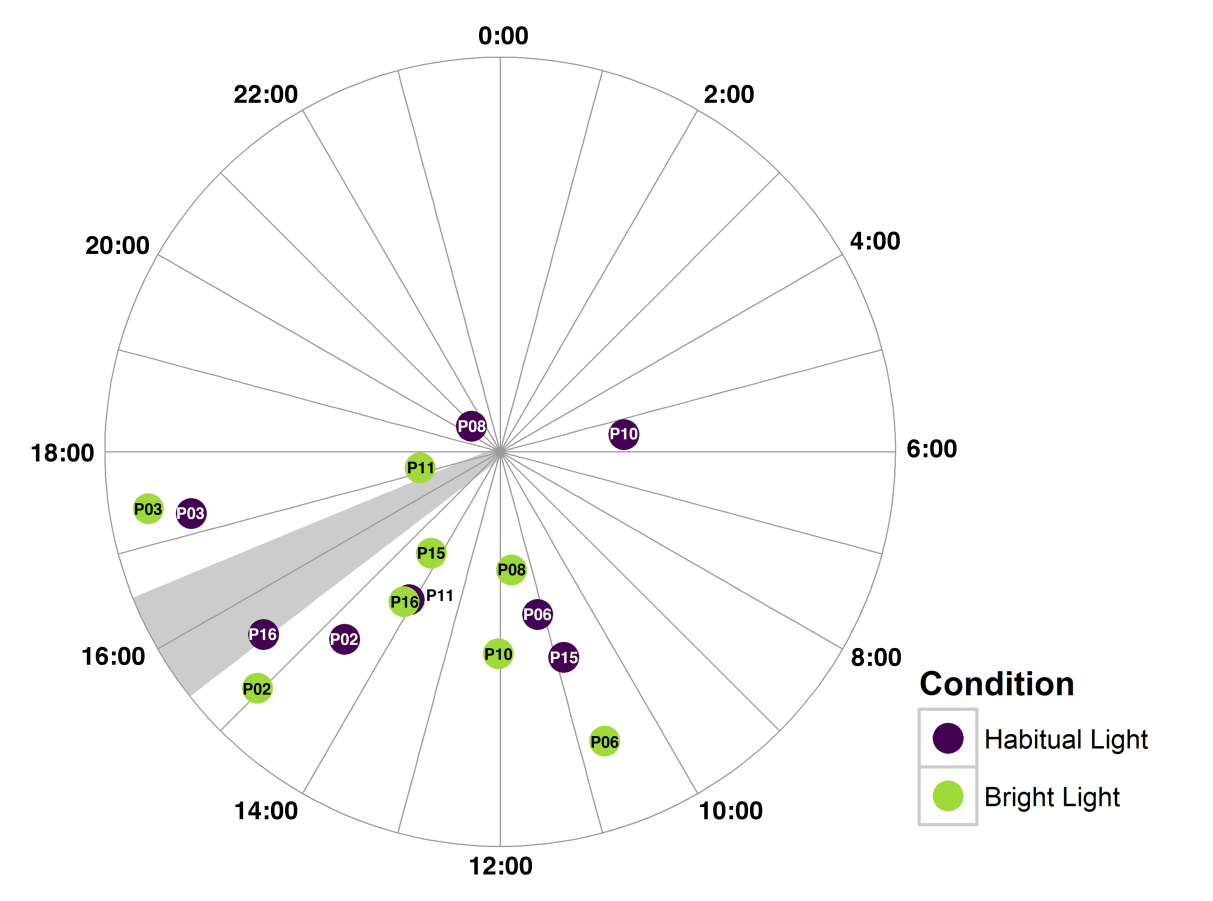
**

**Figure e-3: Times of Occurrence of the Temperature Maximum in the Habitual and the Bright Light Condition.** Each dot represents the time of occurrence of one patient’s temperature maximum in one condition. The distance of each dot from the centre of the plot indicates the normalised power of the circadian peak, i.e. the strength of the circadian rhythm. The right half of the plot represents the time from midnight until noon, the left half the time from noon until midnight. In healthy, day-active individuals the temperature maximum would be expected at about 16:00 hours, the grey area indicates the time period between 15:30 and 16:30 hours.

**Table e-1: Detailed Results of the CRS-R Assessments**

| **Patient ID** | **Experimental Condition** | **Diagnosis HL** | **CRS-R Σ Score HL** | **Diagnosis BL** | **CRS-R Σ Score BL** | **CRS-R**  **Auditory** | | **CRS-R**  **Visual** | | **CRS-R Motor** | | | **CRS-R Oromotor/Verbal** | | **CRS-R Comm-unication** | | | **CRS-R Arousal** | |
| --- | --- | --- | --- | --- | --- | --- | --- | --- | --- | --- | --- | --- | --- | --- | --- | --- | --- | --- | --- |
|  |  |  |  |  |  | **HL** | **BL** | **HL** | **BL** | **HL** | **BL** | **HL** | | **BL** | | **HL** | **BL** | **HL** | **BL** |
| P01 | - | VS/UWS | 6 | - | - | 1 | - | 0 | - | 2 | - | 1 | | - | | 0 | - | 2 | - |
| P02 | 2 | MCS_exit_ | 11 | MCS_exit_ | 11 | 3 | 3 | 0 | 0 | 1 | 1 | 2 | | 2 | | 2 | 2 | 3 | 3 |
| P03 | 2 | VS/UWS | 6 | VS/UWS | 6 | 1 | 1 | 0 | 0 | 2 | 2 | 1 | | 1 | | 0 | 0 | 2 | 2 |
| P04 | - | VS/UWS | 7 | - | - | 1 | - | 1 | - | 2 | - | 1 | | - | | 0 | - | 2 | - |
| P05 | - | VS/UWS | 6 | - | - | 1 | - | 0 | - | 2 | - | 1 | | - | | 0 | - | 2 | - |
| P06 | 2 | VS/UWS | 7 | VS/UWS | 4 | 1 | 0 | 1 | 0 | 2 | 2 | 1 | | 1 | | 0 | 0 | 2 | 1 |
| P07 | - | VS/UWS | 6 | - | - | 1 | - | 0 | - | 2 | - | 1 | | - | | 0 | - | 2 | - |
| ***P08*** | ***1*** | ***VS/UWS*** | ***3*** | ***MCS*** | ***9*** | ***0*** | ***1*** | ***0*** | ***1*** | ***2*** | ***3*** | ***0*** | | ***1*** | | ***0*** | ***1*** | ***1*** | ***2*** |
| P09 | - | VS/UWS | 6 | - | - | 1 | - | 0 | - | 2 | - | 1 | | - | | 0 | - | 2 | - |
| ***P10*** | ***2*** | ***VS/UWS*** | ***7*** | ***MCS*** | ***12*** | ***1*** | ***3*** | ***1*** | ***3*** | ***2*** | ***2*** | ***1*** | | ***1*** | | ***0*** | ***1*** | ***2*** | ***2*** |
| P11 | 2 | VS/UWS | 3 | VS/UWS | 5 | 0 | 0 | 0 | 0 | 2 | 2 | 1 | | 1 | | 0 | 0 | 0 | 2 |
| P12 | - | MCS | 11 | - | - | 2 | - | 3 | - | 2 | - | 1 | | - | | 1 | - | 2 | - |
| P13 | - | VS/UWS | 7 | - | - | 1 | - | 1 | - | 2 | - | 1 | | - | | 0 | - | 2 | - |
| P14 | - | VS/UWS | 1 | - | - | 0 | - | 0 | - | 0 | - | 0 | | - | | 0 | - | 1 | - |
| ***P15*** | ***2*** | ***VS/UWS*** | ***3*** | ***MCS*** | ***7*** | ***0*** | ***0*** | ***0*** | ***3*** | ***1*** | ***1*** | ***1*** | | ***1*** | | ***0*** | ***0*** | ***1*** | ***2*** |
| P16 | 1 | MCS_exit_ | 23 | MCS | 10 | 4 | 2 | 5 | 3 | 6 | 2 | 3 | | 1 | | 2 | 0 | 3 | 2 |
| P17 | - | MCS | 13 | - | - | 2 | - | 3 | - | 4 | - | 1 | | - | | 0 | - | 3 | - |
| P18 | - | MCS | 9 | - | - | 1 | - | 3 | - | 3 | - | 0 | | - | | 0 | - | 2 | - |

Condition: 1 = HL-BL, 2 = BL-HL; Abbreviations: MCS = Minimally Conscious State; VS/UWS = Vegetative State/ Unresponsive Wakefulness Syndrome; TBI = Traumatic Brain Injury; NTBI = Non-Traumatic Brain Injury; Bold and italic font indicates patients with a change of diagnosis between HL and BL conditions.

**Table e-2: Results of the CRS-R Assessments Before and After Light Stimulation**

| **Patient ID** | **Experimental Condition** | **Diagnosis BL** | **CRS-R Σ Score BL_max_** | **Diagnosis BL_pre_** | **CRS-R Σ Score BL_pre_** | **Diagnosis BL_post_** | **CRS-R Σ Score BL_post_** |
| --- | --- | --- | --- | --- | --- | --- | --- |
| P02 | 2 | MCS_exit_ | 11 | MCS_exit_ | 11 | MCS_exit_ | 11 |
| P03 | 2 | VS/UWS | 6 | VS/UWS | 2 | VS/UWS | 6 |
| P06 | 2 | VS/UWS | 4 | VS/UWS | 4 | VS/UWS | 4 |
| ***P08*** | ***1*** | ***MCS*** | ***9*** | ***MCS*** | ***9*** | ***-*** | ***-*** |
| ***P10*** | ***2*** | ***MCS*** | ***12*** | ***MCS*** | ***12*** | ***-*** | ***-*** |
| P11 | 2 | VS/UWS | 5 | VS/UWS | 5 | - | - |
| ***P15*** | ***2*** | ***MCS*** | ***7*** | ***VS/UWS*** | ***4*** | ***MCS*** | ***7*** |
| P16 | 1 | MCS | 10 | MCS | 10 | MCS | 8 |

Please note that the primary aim of this study was to assess the effects of a whole week of bright-light stimulation on patients’ circadian rhythms. Pre to post bright-light stimulation (BLS) changes in CRS-R scores were assessed whenever possible and should inform about the immediate effects of BLS on patients’ behavioural performance. Yet, two CRS-R assessments in such close temporal order are likely to exhaust and fatigue patients, wherefore any changes should be interpreted with caution. Condition: 1 = HL-BL, 2 = BL-HL; Abbreviations: MCS = Minimally Conscious State; VS/UWS = Vegetative State/ Unresponsive Wakefulness Syndrome; TBI = Traumatic Brain Injury; NTBI = Non-Traumatic Brain Injury; BL_max_ = maximal CRS-R score obtained during any of the assessments and the basis for the diagnosis; BL_pre_ = CRS-R score before BL exposure; BL_post_ = CRS-R score after BL exposure. Bold and italic font indicates patients with a change of diagnosis between HL and BL conditions.

**References**

1. Cohen, D. A., Wang, W., Wyatt, J. K., Kronauer, R. E., Dijk, D.-J., Czeisler, C. A., et al. (2010). Uncovering residual effects of chronic sleep loss on human performance. *Science Translational Medicine, 2*(14).

2. Kalmar, K., & Giacino, J. T. (2005). The JFK coma recovery scale - revised. *Neuropsychological Rehabilitation, 15*(3-4), 454-460. doi:10.1080/09602010443000425

3. Hasselberg, M. J., McMahon, J., & Parker, K. (2013). The validity, reliability, and utility of the iButton (R) for measurement of body temperature circadian rhythms in sleep/wake research. *Sleep medicine, 14*(1), 5-11. doi:DOI 10.1016/j.sleep.2010.12.011

4. van Marken Lichtenbelt, W. D., Daanen, H. A., Wouters, L., Fronczek, R., Raymann, R. J., Severens, N. M., et al. (2006). Evaluation of wireless determination of skin temperature using iButtons. *Physiology & Behavior, 88*(4), 489-497.

5. Kräuchi, K., Cajochen, C., Werth, E., & Wirz-Justice, A. (1999). Physiology - Warm feet promote the rapid onset of sleep. *Nature, 401*(6748), 36-37. doi:Doi 10.1038/43366

6. Kräuchi, K., Cajochen, C., Werth, E., & Wirz-Justice, A. (2000). Functional link between distal vasodilation and sleep-onset latency? *American Journal of Physiology - Regulatory, Integrative and Comparative Physiology, 278*(3), R741-R748.

7. R Core Team. (2015). R: A Language and Environment for Statistical Computing. Vienna, Austria: R Foundation for Statistical Computing. Retrieved from <https://www.R-project.org>

8. Kräuchi, K. (2002). How is the circadian rhythm of core body temperature regulated? *Clinical Autonomic Research, 12*(3), 147-149.

9. Aschoff, J., & Wever, R. (1958). Kern und Schale im Wärmehaushalt des Menschen. *Die Naturwissenschaften, 45*.

10. Van Dongen, H. P., Olofsen, E., Van Hartevelt, J. H., & Kruyt, E. W. (1999). Searching for biological rhythms: peak detection in the periodogram of unequally spaced data. *Journal of biological rhythms, 14*(6), 617-620.

11. Lomb, N. R. (1976). Least-squares frequency analysis of unequally spaced data. *Astrophysics and space science, 39*(2), 447-462.

12. Scargle, J. D. (1982). Studies in astronomical time series analysis. II-Statistical aspects of spectral analysis of unevenly spaced data. *The Astrophysical Journal, 263*, 835-853.

13. Ruf, T. (1999). The Lomb-Scargle periodogram in biological rhythm research: analysis of incomplete and unequally spaced time-series. *Biological Rhythm Research, 30*(2), 178-201.

14. Czeisler, C. A., Duffy, J. F., Shanahan, T. L., Brown, E. N., Mitchell, J. F., Rimmer, D. W., et al. (1999). Stability, precision, and near-24-hour period of the human circadian pacemaker. *Science, 284*(5423), 2177-2181.

15. Blume, C., Santhi, N., & Schabus, M. (2016). ‘nparACT’package for R: A free software tool for the non-parametric analysis of actigraphy data. *MethodsX*.

16. Burchett, W. W., Ellis, A. R., Harrar, S. W., & Bathke, A. C. (2017). Nonparametric Inference for Multivariate Data: The R Package npmv. *Journal of Statistical Software, 76*(4), 18. doi:10.18637/jss.v076.i04

17. McLeod, A. I. (2011). Kendall: Kendall rank correlation and Mann-Kendall trend test. Retrieved from <https://CRAN.R-project.org/package=Kendall>

18. Noguchi, K., Gel, Y. R., Brunner, E., & Konietschke, F. (2012). nparLD: An R Software Package for the Nonparametric Analysis of Longitudinal Data in Factorial Experiments. *Journal of Statistical Software, 50*(12), 1-23.
